# *PPP2R1A* regulated by PAX3/FOXO1 fusion contributes to the acquisition of aggressive behavior in PAX3/FOXO1-positive alveolar rhabdomyosarcoma

**DOI:** 10.18632/oncotarget.25392

**Published:** 2018-05-18

**Authors:** Keisuke Akaike, Yoshiyuki Suehara, Shinji Kohsaka, Takuo Hayashi, Yu Tanabe, Saiko Kazuno, Kenta Mukaihara, Midori Toda-Ishii, Taisei Kurihara, Youngji Kim, Taketo Okubo, Yasuhide Hayashi, Kazuya Takamochi, Fumiyuki Takahashi, Kazuo Kaneko, Marc Ladanyi, Tsuyoshi Saito

**Affiliations:** ^1^ Department of Orthopedic Surgery, Juntendo University School of Medicine, Tokyo, Japan; ^2^ Department of Human Pathology, Juntendo University School of Medicine, Tokyo, Japan; ^3^ Department of Medical Genomics, Graduate School of Medicine, The University of Tokyo, Tokyo, Japan; ^4^ Laboratory of Proteomics and Biomolecular Science, Research Support Center, Juntendo University Graduate School of Medicine, Tokyo, Japan; ^5^ Department of Hematology/Oncology, Gunma Children's Medical Center, Shibukawa, Gunma, Japan; ^6^ Department of General Thoracic Surgery, Juntendo University School of Medicine, Tokyo, Japan; ^7^ Department of Respiratory Medicine, Juntendo University School of Medicine, Tokyo, Japan; ^8^ Department of Pathology, Memorial Sloan-Kettering Cancer Center, New York, NY, USA

**Keywords:** alveolar rhabdomyosarcoma (ARMS), proteomics, PAX3/FOXO1, PPP2R1A, PP2A

## Abstract

To better characterize the oncogenic role of the *PAX3-FOXO1* fusion protein in the acquisition of aggressive behavior in ARMS, we employed a proteomic approach using a PAX3-FOXO1 knockdown system in ARMS cell lines. This approach revealed a protein list consisting of 107 consistently upregulated and 114 consistently downregulated proteins that were expected to be regulated by PAX3-FOXO1 fusion protein. Furthermore, we identified 16 upregulated and 17 downregulated critical proteins based on a data-mining analysis. We also evaluated the function of PPP2R1A in ARMS cells. The *PPP2R1A* expression was upregulated at both the mRNA and protein levels by *PAX3-FOXO1* silencing. The silencing of *PPP2R1A* significantly increased the cell growth of all four ARMS cells, suggesting that PPP2R1A still has a tumor suppressive function in ARMS cells; however, the native expression of PPP2R1A was low in the presence of PAX3-FOXO1. In addition, the activation of PP2A—part of which was encoded by *PPP2R1A*—by FTY720 treatment in ARMS cell lines inhibited cell growth. On the human phospho-kinase array analysis of 46 specific Ser/Thr or Tyr phosphorylation sites on 39 selected proteins, eNOS, AKT1/2/3, RSK1/2/3 and STAT3 phosphorylation were decreased by FTY-720 treatment. These findings suggest that PPP2R1A is a negatively regulated by PAX3-FOXO1 in ARMS. The activation of PP2A—probably in combination with kinase inhibitors—may represent a therapeutic target in ARMS. We believe that the protein expression profile associated with PAX3-FOXO1 would be valuable for discovering new therapeutic targets in ARMS.

## INTRODUCTION

Rhabdomyosarcoma (RMS), a high-grade tumor of soft-tissue, is divided into four subtypes: embryonal RMS (ERMS), alveolar RMS (ARMS), pleomorphic RMS and spindle cell/sclerosing RMS [1]. Among these tumors, two types of chromosomal translocations, t(2;13)(q35;q14) and t(1:13)(p36;q14), have been detected in approximately 70–80% of ARMS resulting in the formation of either *PAX3-FOXO1* or *PAX7-FOXO1* fusion, respectively [2]. Fusion-positive ARMSs show a worse prognosis than fusion-negative ARMSs, and ARMSs with the *PAX3-FOXO1* variant have a poorer outcome than those with *PAX7–FOXO1* [2–5]. These PAX3-FOXO1/PAX7-FOXO1 fusion proteins are critical transcriptional factors and are thereby considered to have a central role in the pathogenesis of ARMS; however, the functions of these fusion proteins are unclear. Several gene expression studies have identified potential relevant genes in PAX3-FOXO1-positive-ARMS [6–9]. However, the protein expression signatures associated with these fusion proteins have not been clearly demonstrated.

The serine-threonine protein phosphatase 2A (PP2A) regulates multiple cell signaling cascades and its inactivation by viral oncoproteins, the mutation of specific structural subunits, or the upregulation of the cellular endogenous inhibitors may contribute to malignant transformation by regulating specific phosphorylation events [10]. PP2A dysfunction has been implicated in various malignancies [11]. We recently demonstrated that mutations of the *PPP2R1A* encoding part of PP2A frequently occurred in gastrointestinal stromal tumors (GISTs) and that these mutations were associated with a poorer prognosis in GISTs [12]. Furthermore, the inactivation of PP2A by these mutations was associated with the increased phosphorylation of its substrates in GISTs [12]. The pharmacological modulation of PP2A activity is becoming an attractive strategy for cancer treatment [10]. Some compounds targeting PP2A, such as FTY720, are able to induce PP2A reactivation and subsequent cell death in several types of cancer [10, 13–15].

In the present study, to better characterize the oncogenic role of the PAX3-FOXO1 fusion protein in the acquisition of aggressive behavior in ARMS, we conducted proteomic studies using a *PAX3-FOXO1* knockdown system in ARMS cell lines and *in vitro* assays, and found that among the protein list, proteins encoded by *PPP2R1A* were downregulated in the native ARMS cell lines. The expression of *PPP2R1A* encoding alpha-subunit of PP2A was upregulated by the transfection of siRNAs against *PAX3-FOXO1* at the mRNA and protein expression levels. Furthermore, the cell growth of ARMS cells was drastically increased by the transfection of siRNAs against *PPP2R1A*. In addition, the activation of PP2A by FTY720 treatment in ARMS cell lines inhibited cell growth. Taken together, *PPP2R1A* is negatively regulated by PAX3-FOXO1 fusion protein in ARMS and the activation of PP2A may represent a new therapeutic target in ARMS.

## RESULTS

### Candidate proteins associated with the PAX3-FOXO1 fusion product in ARMS

**Figure 1:**
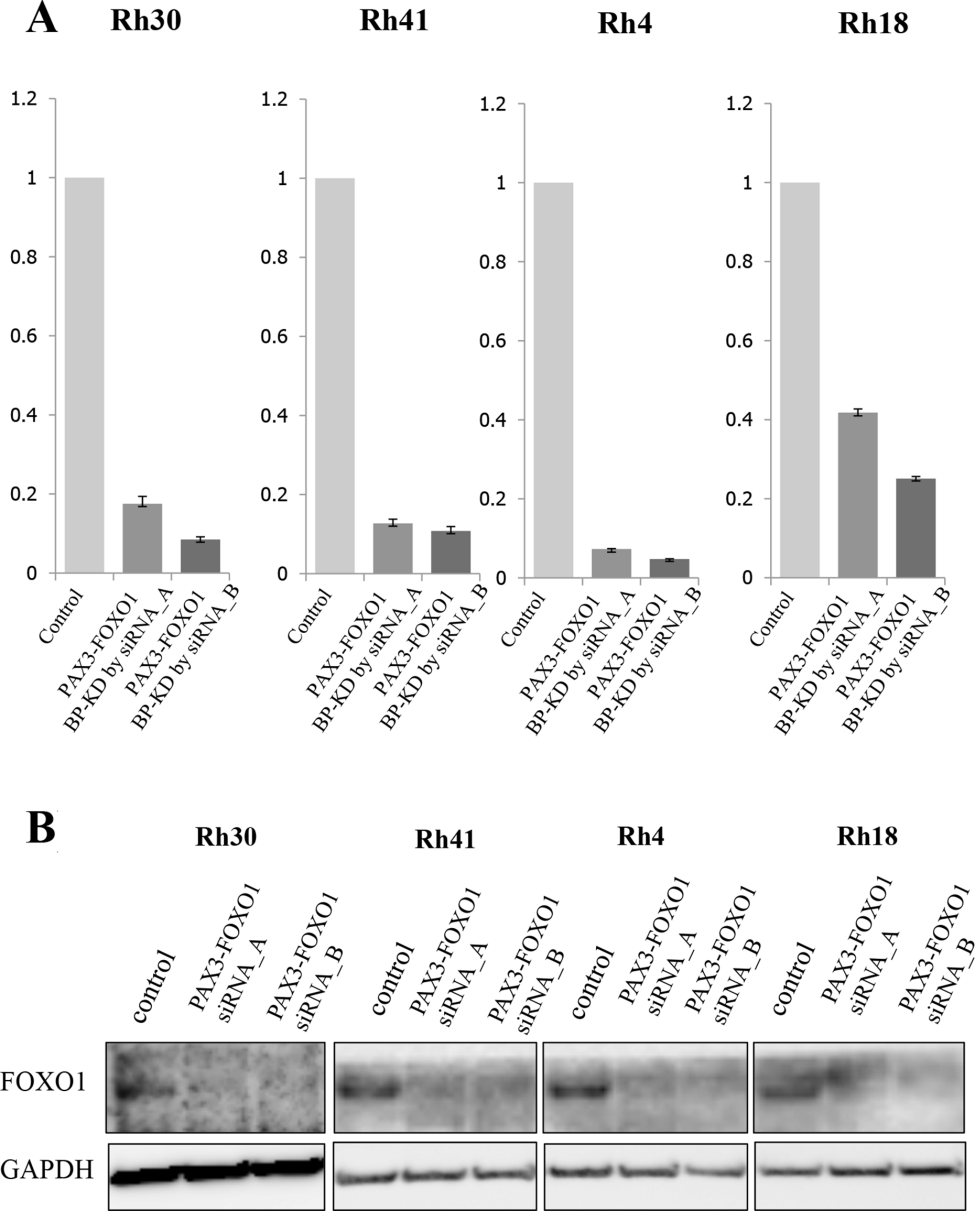
The expression of PAX3-FOXO1 and siRNA targeting PAX3-FOXO1 in ARMS cell lines (**A**) Proteomic studies were performed using proteins extracted from four ARMS cell lines (Rh4, Rh18, Rh30 and Rh41) that were transfected with siRNAs targeting the PAX3-FOXO1 break point (BP). The quantitative PCRs (qPCRs) showed that PAX3-FOXO1 BP siRNA inhibited the mRNA expression of PAX3-FOXO1 in all four ARMS cell lines. (**B**) The protein expression of FOXO1 was verified by Western blotting. Western blotting of FOXO1 showed that the cell lines with PAX3-FOXO1 siRNA knockdown had lower expression levels of FOXO1 in comparison to control cells in the four ARMS cell lines.

Four ARMS cell lines were transfected with 2 different siRNA targeted for *PAX3-FOXO1* and incubated after transfection and collected after 72 h. The suppression of the fusion gene transcript was confirmed by a quantitative reverse transcription-PCR (Figure 1A). In this knockdown assay, the growth of all ARMS cell lines was decreased, following the knockdown of *PAX3-FOXO1,* to approximately 60% of that in the control groups (Figure 1A and Figure 1B). Proteins derived from each transfected ARMS cell line were analyzed using the i-TRAQ method and 8 distinct protein profiles were observed, in which 1,300–2,400 proteins showed altered expression levels (Supplementary Tables 1–8). In order to narrow down the candidate proteins, we selected proteins that were significantly altered in at least 2 of the 4 ARMS cell lines. This approach narrowed down the protein lists to 107 consistently upregulated and 114 consistently downregulated proteins (Supplementary Table 9). These upregulated or downregulated protein profiles were further analyzed using the Oncomine and IPA databases. Finally, a protein list consisting of 16 upregulated and 17 downregulated proteins that were expected to be regulated by PAX3-FOXO1 fusion protein was obtained (Table 1). Among these, we focused on PPP2R1A to test the biological significance in the setting of ARMS.

**Table 1:** List of proteins related to the PAX3/FOXO3 fusion gene, determined based on data-mining

Accession No.	Symbol	Protein name	Up/Down	Fold difference	*P*-value
P80297	MT1X	Metallothionein-1X	Down	0.47	3.29E-02
P42766	RPL35	60S ribosomal protein L35	Down	0.57	1.98E-02
O75531	BANF1	Barrier-to-autointegration factor	Down	0.58	2.49E-02
P62753	RPS6	40S ribosomal protein S6	Down	0.66	1.91E-02
P61254	RPL26	60S ribosomal protein L26	Down	0.68	9.97E-03
P22087	FBL	rRNA 2'-O-methyltransferase fibrillarin	Down	0.70	3.18E-02
P00367	GLUD1	Glutamate dehydrogenase 1, mitochondrial	Down	0.71	2.79E-02
Q02878	RPL6	60S ribosomal protein L6	Down	0.72	1.74E-02
Q15233	NONO	Non-POU domain-containing octamer-binding protein	Down	0.76	3.26E-02
P62633	CNBP	Cellular nucleic acid-binding protein	Down	0.76	3.75E-02
P07900	HSP90AA1	Heat shock protein HSP 90-alpha	Down	0.78	1.95E-03
P08107	HSPA1A	Heat shock 70 kDa protein 1A/1B	Down	0.81	1.50E-04
P06753	TPM3	Isoform 2 of Tropomyosin alpha-3 chain	Down	0.81	1.54E-02
Q99615	DNAJC7	DnaJ homolog subfamily C member 7	Down	0.83	4.76E-02
P17987	TCP1	T-complex protein 1 subunit alpha	Down	0.84	2.25E-02
P48643	CCT5	T-complex protein 1 subunit epsilon	Down	0.86	1.80E-02
P13639	EEF2	Elongation factor 2	Down	0.93	4.50E-02
Q14204	DYNC1H1	Cytoplasmic dynein 1 heavy chain 1	Up	1.17	1.47E-02
P51858	HDGF	Hepatoma-derived growth factor	Up	1.19	1.72E-02
P12956	XRCC6	X-ray repair cross-complementing protein 6	Up	1.24	2.25E-02
Q13098	GPS1	COP9 signalosome complex subunit 1	Up	1.27	2.49E-02
Q9BSJ8	ESYT1	Extended synaptotagmin-1	Up	1.28	2.75E-03
P15121	AKR1B1	Aldose reductase	Up	1.29	1.58E-02
Q14315	FLNC	Filamin-C	Up	1.31	7.14E-04
P07237	P4HB	Protein disulfide-isomerase	Up	1.32	7.10E-03
P08758	ANXA5	Annexin A5	Up	1.32	2.37E-02
P30153	PPP2R1A	Serine/threonine-protein phosphatase 2A 65 kDa regulatory subunit A alpha isoform	Up	1.37	1.60E-02
P37268	FDFT1	Squalene synthase	Up	1.43	4.10E-03
P40123	CAP2	Adenylyl cyclase-associated protein 2	Up	1.43	6.33E-03
P52209	PGD	6-phosphogluconate dehydrogenase, decarboxylating	Up	1.47	3.38E-03
O00299	CLIC1	Chloride intracellular channel protein 1	Up	1.52	1.54E-02
P08962	CD63	CD63 antigen	Up	1.59	2.76E-02
P07108	DBI	Acyl-CoA-binding protein	Up	1.64	4.10E-02

### *PPP2R1A* inhibition by siRNA significantly promotes cell proliferation in ARMS cell lines

**Figure 2:**
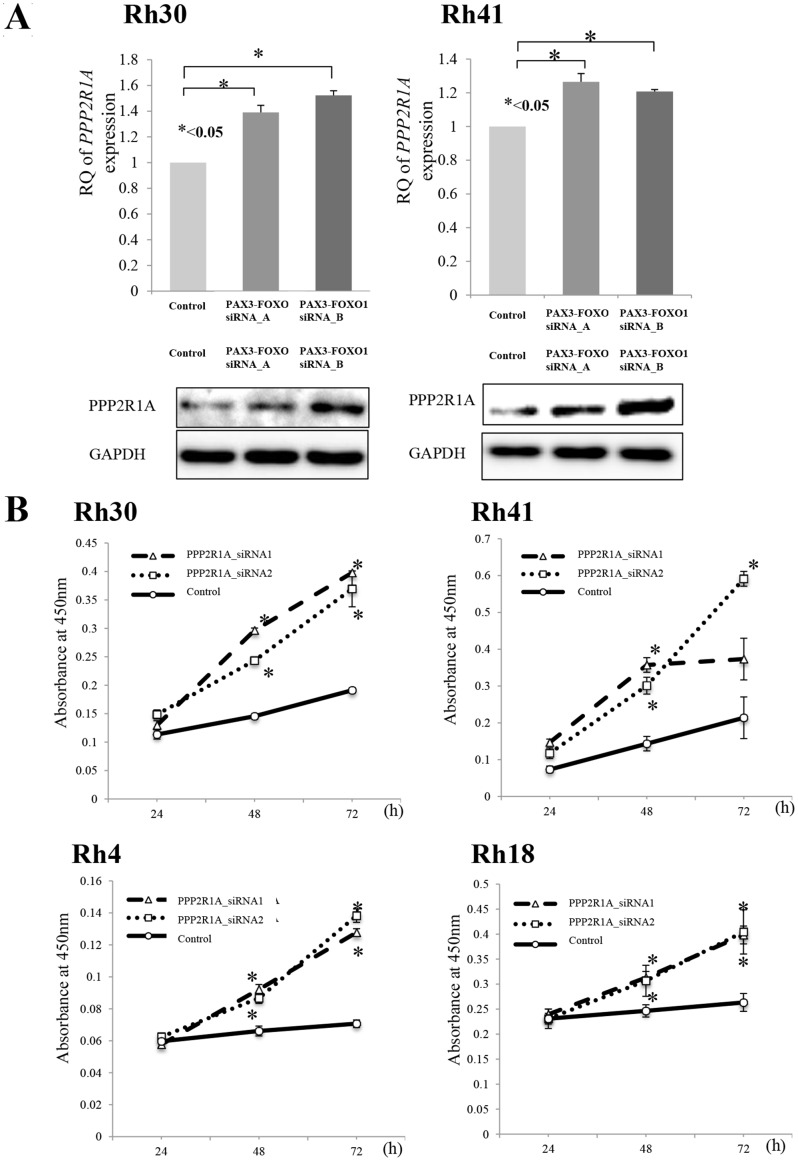
(**A**) Silencing PAX3-FOXO1 activated the expression of PPP2R1A and (**B**) cell viability following PPP2R1A knockdown in ARMS cell lines. (A) To investigate the association between PAX3-FOXO1 and PPP2R1A, siRNA PAX3-FOXO1 was performed and the mRNA and protein expression levels were measured by both a q-PCR and Western blotting. The silencing of PAX3-FOXO1 activated the expression of PPP2R1A in ARMS cell lines (Rh31 and Rh40); this was confirmed by both the mRNA and protein levels. (B) PPP2R1A siRNA knockdown in ARMS cell lines (Rh31 and Rh40) was performed to verify the associations between the PPP2R1A expression and cell viability. PPP2R1A siRNA suppressed the expression of PPP2R1A in ARMS cell lines and the silencing of PPP2R1A activated the cell viability in all four ARMS cell lines.

Before characterizing the role of PPP2R1A encoded by *PPP2R1A* in RMS cells, we confirmed the proteomics findings. *PAX3-FOXO1* silencing upregulated the expression of *PPP2R1A* at both the mRNA and protein levels (Figure 2A). Next, we performed cell proliferation assays in ARMS cell lines using siRNA against *PPP2R1A*. This silencing of *PPP2R1A* significantly increased the cell growth of all ARMS cells (Figure 2B). These findings suggest that PPP2R1A has a tumor suppressive function in ARMS cells; however, the native expression of PPP2R1A was low in the presence of PAX3-FOXO1.

### FTY720, a PP2A-activating drug, significantly inhibits the cellular proliferation of ARMS cell lines

**Figure 3:**
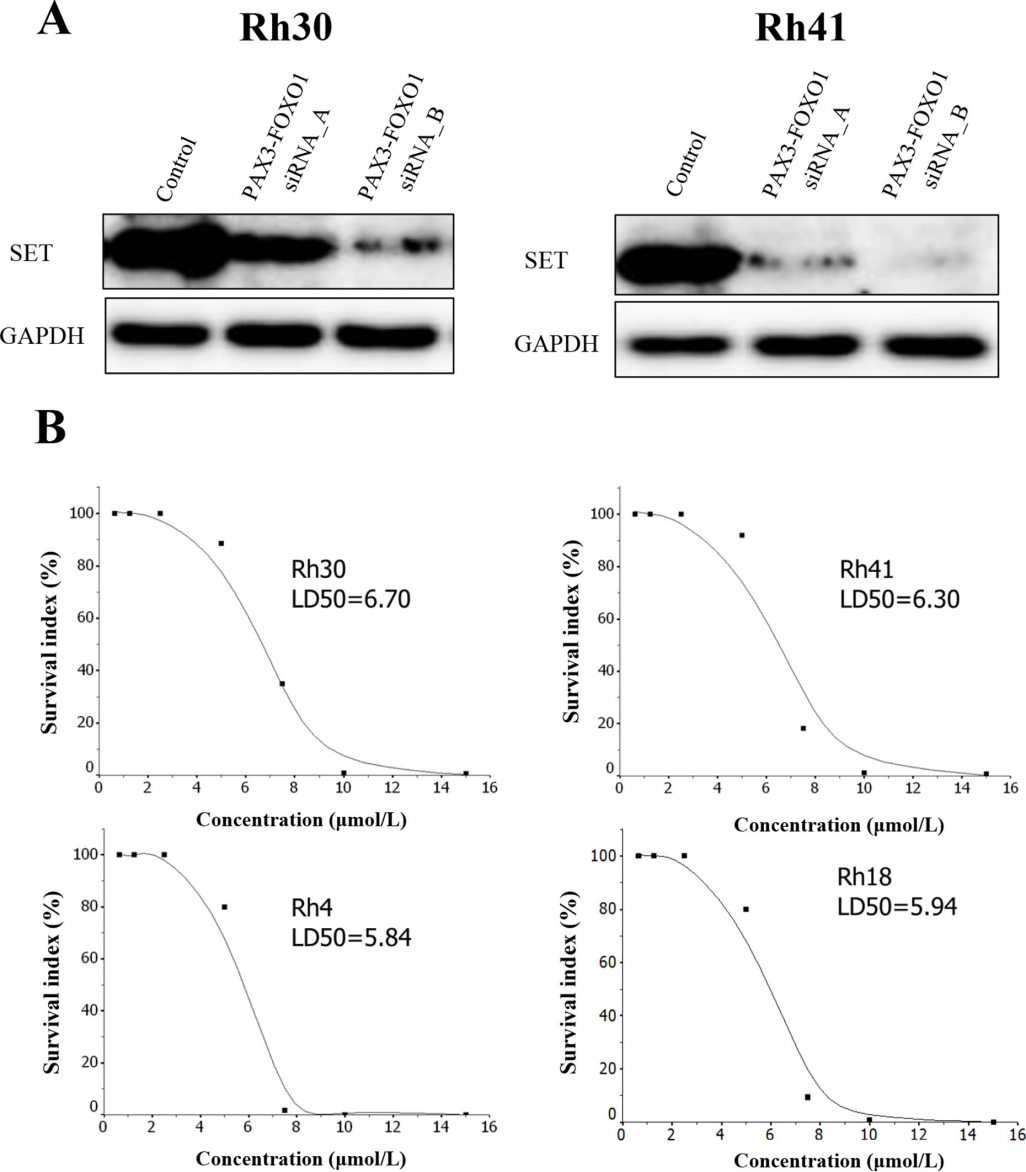
(**A**) The silencing of PAX3-FOXO1 suppressed the expression of SET and (**B**) the cell viability curve of FTY720 in 4 ARMS cell lines. (A) PAX3-FOXO1 siRNA knockdown in ARMS cell lines (Rh31 and Rh40) was performed to verify the association between the expression of PAX3-FOXO1 and SET. PAX3-FOXO1 siRNA suppressed the expression of SET in the two ARMS cell lines; this was confirmed by Western blotting. (B) The cell viability of FTY720 was investigated in four ARMS cell lines. FTY720 significantly inhibited the cell viability in all ARMS cell lines in a dose-dependent manner.

Because it has been shown that FTY-720 reactivates PPP2R1A via SET that inhibits the function of PP2A [16], we first confirmed that SET was strongly expressed in ARMS cells (Figure 3A). We then checked whether the expression of SET could be affected by the *PAX3-FOXO1* inhibition in ARMS cells. This experiment demonstrated that the SET expression significantly decreased according to the knockdown of *PAX3-FOXO1* fusion (Figure 3A). This result also confirmed accuracy of our proteomic studies, as our proteomic profiles, obtained by silencing *PAX3-FOXO1*, also identified SET as one of the down regulated proteins (Supplementary Table 9). Next, we tried to see whether FTY720, a PP2A-activating drug, could affect the cellular proliferation in ARMS cells. This assay suppressed the cell growth in a dose-dependent manner in all ARMS cell lines and proved that FTY720 was effective under the presence of PAX3-FOXO1 fusion in ARMS (Figure 3B).

### Human phospho-kinase array analysis

To identify the cellular signaling pathways that were activated in association with FTY-720 in ARMS cells, we performed a human phospho-kinase array analysis of 46 specific Ser/Thr and Tyr phosphorylation sites on 39 selected proteins. eNOS, AKT1/2/3, RSK1/2/3 and STAT3 phosphorylation were significant decreased following FTY-720 treatment (<55%) (Supplementary Figure 1).

## DISCUSSION

Most ARMSs are characterized by chromosomal translocation—either t(2;13)(q35;q14) or t(1:13)(p36;q14)—leading to the formation of *PAX3-FOXO1* or *PAX7-FOXO1* fusion, respectively. It has been suggested that these PAX3-FOXO1/PAX7-FOXO1 fusion proteins have a central role in the pathogenesis of ARMS; however, the function of these fusion proteins remains unclear. In this regard, the previous studies demonstrated that PAX3-FOXO1 alone was not sufficient for tumorigenesis and that additional changes, such as increased MYCN and the expression of TERT were required [17, 18]. In addition, PAX3-FOXO1 alone did not transform immortalized human myoblasts in culture, and induced slow-growing tumors in immunocompromised mice [3, 19]. However, the combination of PAX3-FOXO1 and MYCN transformed the myoblasts and induced rapid tumorigenesis [19]. In this study, the knockdown of PAX3-FOXO1 in ARMS cell lines reduced the cell proliferation to approximately 60% in comparison to the control, which was partly in line with the previous reports suggesting that PAX3-FOXO1 alone is not responsible for the proliferative advantage. In sarcoma cell lines, the silencing of EWS-Fli1 [20] and PAX3-FOXO1 [21] induces the suppression of cell growth to almost the same level; however, the knockdown of *KIT* in GIST cells was associated with strong growth suppression (unpublished data). This growth inhibitory effect of gene silencing in ARMS seems to be insufficient for a single therapeutic target, and the drug delivery problem remains to be solved in this system. We therefore conducted a proteomic analyses of ARMS to characterize the protein profile associated with *PAX3-FOXO1* fusion with cross-reference to the gene expression data, with the aim of identifying efficient therapeutic targets in this tumor that could be treated using easy methods of administration (*i.e.,* oral administration). With this background, we focused on the *PPP2R1A* coding part of PP2A as a possible new therapeutic target in ARMSs. In this study, we found that phosphatase activity by PP2A was strongly repressed in this tumor; that is, the PPP2R1A expression was suppressed and that expression of SET, a PP2A suppressor, was upregulated by PAX3-FOXO1 in ARMS. Additionally, in our proteomic profiles regarding suppressing of *PAX3-FOXO1*, we successfully identified SET protein as a down regulated protein (Supplementary Table 9). Therefore, we believe that this concordance also indicated accuracy in our proteomic analyses.

PP2A consists of several isoforms, including structure (A) subunits, regulatory (B) subunits, and catalyst (C) subunits [22–24]. The regulatory (B) subunit has several subunit types [22–24]. Previous articles have revealed that genomic mutations in the Aα subunit of PP2A in human tumors interferes with the binding to other B and C subunits [22–24]. With respect to the associations among these A, B, and C subunits in expression, previous reports also revealed that the levels of the B and C subunits of PP2A were markedly reduced while the PP2A-Aα expression was suppressed [22]. This was because both the B and C subunits of PP2A were unstable unless bound to the A subunit PP2A [22]. Therefore, previous studies regarding the PP2A functions concluded that the expression of PPP2R1A were suppressed by PAX3/FOXO1 siRNA in ARMS cell lines might lead to lower expression of both the B and C subunits of PP2A.

FTY720 is an immunomodulatory agent used as an oral therapy for multiple sclerosis [22]; it is also a PP2A-activating drug [16]. FTY720 undergoes phosphorylation (FTY720-P) by sphingosine kinase 2 (SPHK2) to act as an immunosuppressant, and binds/internalizes the sphingosine-1-phosphate receptor (S1PR1) [25]. FTY720 also selectively induces apoptosis of neoplastic but not normal cells [11]. This anticancer activity does not require phosphorylation but mostly depends on its ability to activate PP2A [11]. The anticancer activity of FTY720 depends on the interaction/sequestration of the PP2A inhibitor SET; however, conversion into FTY720-P is not required [16]. In this study, the addition of FTY720 to ARMS cells drastically suppressed the cell growth in all 4 ARMS cell lines in a dose-dependent manner, confirming that SET surely disturbs the function of PP2A in ARMS. In addition to the expression of *PPP2R1A*. which encodes PP2A, we could confirm that SET is strongly expressed in ARMS cells and that the expression of SET in ARMS cell lines also decreased by the knockdown of *PAX3-FOXO1*. This finding suggests that the expression of SET is also positively regulated by PAX3-FOXO1 in ARMS. Because it has been shown that SET inhibits the function of PP2A, the reduced activity of PP2A is associated with not only the reduced expression of PP2A itself but also with the increased expression of SET in *PAX3-FOXO1*-positive-ARMS. These findings suggest that the function of PP2A in ARMS is strongly suppressed by *PAX3-FOXO1* fusion.

The human phospho-kinase array analysis after FTY-720 treatment revealed eNOS, AKT1/2/3, RSK1/2/3 and STAT3 as a substrate of PP2A. A recent study revealed that AKT1/2/3 and PAX3-FOXO1 cooperation enforces myogenic differentiation blockade as a cause of differentiation failure in ARMS [26]. Furthermore, a study reported that STAT3 supported the growth and survival of RMS cells [27]. With regard to eNOS, Cunha *et al*. demonstrated that sarcomas frequently expressed eNOS [28]. A previous report found that mTOR inhibitor (rapamycin) inhibits insulin-like growth factor 1 (IGF-1)-stimulated cell motility through the PP2A pathway [29]. Furthermore, those functional studies showed that rapamycin inhibited the basal or IGF-1-induced motility of Ewing sarcoma (Rh1) and ARMS (Rh30) cell lines [29]. In addition, the treatment of cells with rapamycin activated the PP2A activity and concurrently inhibited the IGF-1 stimulated phosphorylation of several critical pathways [29]. Based on our findings and those of previous studies, combination therapy with FYT720 and other anticancer agents, including kinase inhibitors, would be beneficial to ARMS patients.

Finally, we believe that these protein signatures regulated by PAX3-FOXO1 will help to elucidate the malignant progression of ARMS with *PAX3-FOXO1* fusion and lead to the development of novel therapeutic strategies. However, we are aware that this approach cannot identify proteins dysregulated by genetic alterations such as mutation and amplification, which are not expected to be regulated by fusion protein. For example, previous studies show that the expression of anaplastic lymphoma kinase (ALK) in RMS is frequently observed in alveolar RMS with *PAX3-FOXO1* fusion and that the expression of ALK is associated with adverse clinical outcomes [30–33]. The high expression of ALK in ARMS with *PAX3-FOXO1* fusion has been shown to be due to gene amplification [30, 31, 33]. However, the ALK protein level was not affected by the manipulation of the fusion gene in ARMS cells with *PAX3-FOXO1* fusion.

In summary, the downregulation of *PPP2R1A* by PAX3-FOXO1 fusion protein plays an important role in the acquisition of malignant potential in ARMS cells with *PAX3-FOXO1* fusion. In addition, strong expression of SET, PP2A inhibitor, positively regulated by PAX3-FOXO1 fusion protein also contributes to the dysfunction of phosphatase activity and would be involved in the acquisition of aggressive behavior of ARMS in a coordinated manner.

## MATERIALS AND METHODS

### Cell lines

We used 4 ARMS cell lines (Rh4, Rh18, Rh30 and Rh41) with *PAX3-FOXO1* fusion cultured in DMEM with 10% FBS. Rh30 and Rh41were kindly provided by Dr. Peter Houghton (The Research Institute at Nationwide Children's Hospital, Columbus, OH, USA). Rh4 and Th18 were obtained from Dr. Thomas Look (Dana-Farber Cancer Institute, Boston, MA, USA).

### Knockdown of PAX3-FOXO1 in ARMS cell lines

To evaluate the function of the endogenous PAX3-FOXO1 fusion protein in ARMS, we performed RNA interference using siRNA duplexes against *PAX3-FOXO1*. Briefly, for preparation of the cell line, 24h before transfection, cells at 80% confluence were trypsinized and diluted with fresh medium, without antibiotics, to 3 × 10^5^ cells/ml, and were then transferred into either 6-well plates (2.5 ml/well) or a 96-well plate (0.1 ml/well). Transfection of 2 different siRNAs for each target of *PAX3-FOXO1* break point: *PAX3-FOXO1_1* (s: GCCUCUCACCUCAGAAUUCdTdT, as: GAA UUCUGAGGUGAGAGGCdTdT, Sigma-Aldrich, MO, USA) and PAX3-FOXO1_2 (s: CCUCUCACCUCAGA AUUCAdTdT, as: UGAAUUCUGAGGUGAGAGGdTdT, Sigma-Aldrich, MO, USA) [21], *PPP2R1A* (SASI_Hs01_00206464, SASI_Hs01_00206465, Sigma-Aldrich MO, USA), and a scrambled siRNA as a negative control (Sigma-Aldrich MO, USA) was carried out using Lipofectamine™ RNAiMAX reagent (Thermo Fisher Scientific, CA, USA) and 30 pmol of each siRNA duplex. Cells were harvested at 24, 48, 72, and 96 h after transfection, then subjected to further analyses, including a proteomic analysis, Western blotting, a proliferation assay, and a real-time quantitative PCR.

### Proteomic analysis by i-TRAQ

In order to identify the protein signatures regulated by the PAX3-FOXO1 fusion gene product in ARMS and to elucidate the function of PAX3-FOXO1, we performed proteomic analyses using isobaric tags for the relative and absolute quantitation (i-TRAQ) and determined the protein profiles regulated by PAX3-FOXO1 [34, 35].

### RNA extraction and real-time PCR

Total RNA was extracted from cell pellets harvested at 72 h after transfection using TRIzol Reagent (Gibco/BRL, Tokyo, Japan) according to the manufacturer's protocol. Five micrograms of RNA of each sample were used for the subsequent reverse transcription reaction (SuperScriptII) (Thermo Fisher Scientific, CA, USA). A semi-quantitative PCR was performed for *PAX3-FOXO1* and *PPP2R1A* using a StepOne Real-Time PCR System (Applied Biosystems, CA, USA) and the predeveloped TaqMan assay reagents for *PPP2R1A* (Hs00204426_m1, Applied Biosystems CA, USA) and *PAX3-FOXO1* (Hs03024825_ft, Applied Biosystems CA, USA). Human *TBP* was used as an endogenous control (Human *TBP* Endogenous Control, 4333769F, Applied Biosystems CA, USA). The comparative C_T_ (ΔΔC_T_) method was used for the semi-quantification of the PCR samples.

### Cell proliferation assay

Cells (1 × 10^6^) of each cell line were plated in a 100-mm-diameter culture dish with 8 ml of RPMI supplemented with 10% calf serum and antibiotics (SM and PC). The number of cells was counted after 24 h, 72 h, and 120 h using a TC20 Automated Cell Counter (BIO-RAD); quantification was performed in triplicate.

### Western blotting

The proteins were extracted from four ARMS cells and were separated via SDS-PAGE and transferred to nitrocellulose membranes. The membranes were incubated with either of the following antibodies: rabbit polyclonal antibodies against PPP2R1A (dilution 1:200, Santa Cruz, sc-168), SET (dilution 1: 200, Santa Cruz, sc-18076), mouse monoclonal antibody against GAPDH (dilution 1: 500, Santa Cruz, sc-32233) and FOXO1 (dilution 1: 100, Santa Cruz, sc-374427). After incubation, the membranes were washed three times with Tris-EDTA buffer and then reacted with horseradish peroxidase-conjugated secondary antibodies (1:1,000 dilution, GE Healthcare Biosciences).

### Human phospho-kinase array analysis

We performed a human phospho-kinase array analysis using a RMS cell line (Rh31). The relative phosphorylation levels of 39 selected proteins on the array were acquired using a Proteome Profiler Human Phospho-Kinase Array Kit (R&D Systems, Minneapolis, MN, USA) according to the manufacturer's instructions. The expression levels of phosphorylation proteins were quantified using the Fuji Film Multi Gauge software program (Tokyo, Japan).

### Statistical analysis

The Mann–Whitney *U* test were used to examine differences in cellular proliferation and gene expression levels according to the knockdown of each target gene.

## SUPPLEMENTARY MATERIALS FIGURES AND TABLES




















