# Supplementary material for: PPP2R1A regulated by PAX3/FOXO1 fusion contributes to the acquisition of aggressive behavior in PAX3/FOXO1-positive alveolar rhabdomyosarcoma
Source: Oncotarget. 2018 May 18;9(38):25206–15. doi: 10.18632/oncotarget.25392 (PMC5982774; doi:10.18632/oncotarget.25392)
Supplement: Supplementary file 1 [file oncotarget-09-25206-s001.pdf]

PPP2R1A regulated by PAX3/FOXO1 fusion contributes to the acquisition of aggressive behavior in PAX3/FOXO1-positive alveolar rhabdomyosarcoma

SUPPLEMENTARY MATERIALS

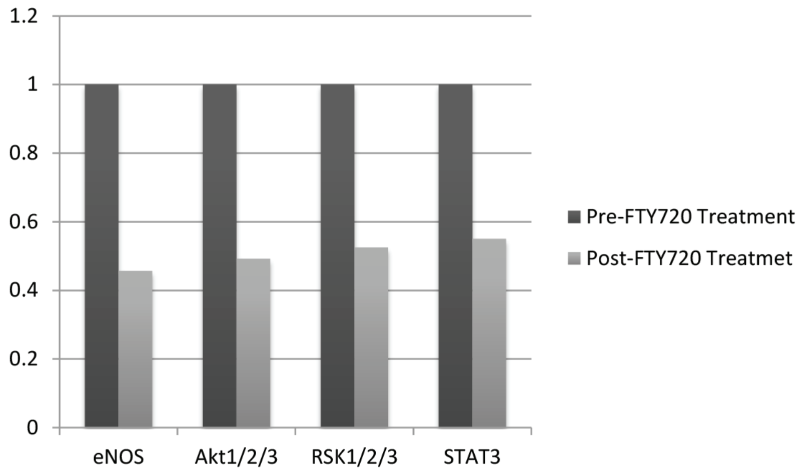

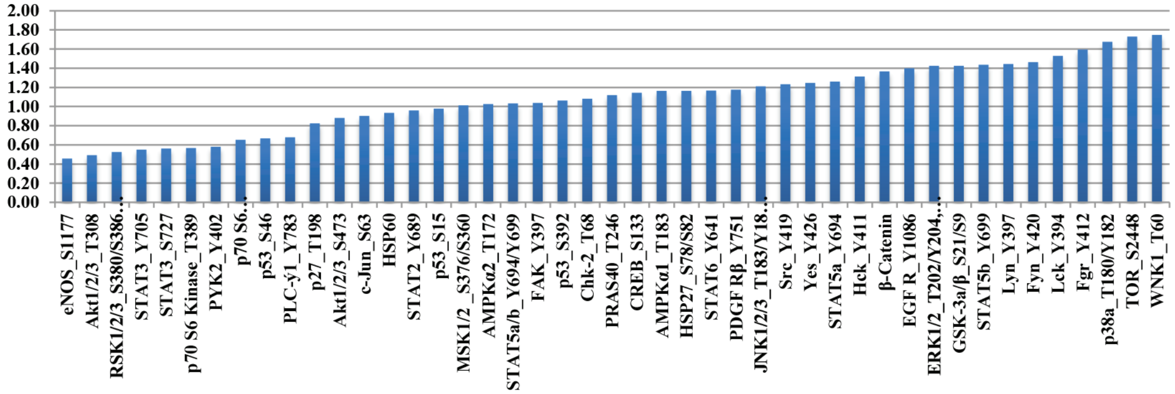

Supplementary Figure 1: The cellular signaling pathways associated with FTY-720. To identify the cellular signaling pathways activated in association with FTY-720 in ARMS cells, a human phospho-kinase array analysis consisting of 46 specific Ser/Thr or Tyr phosphorylation sites on 39 selected proteins was performed to compare FTY-720 treatment with non-treatment. The array analyses showed that eNOS, AKT1/2/3, RSK1/2/3 and STAT3 phosphorylation in the FTY-720 treatment were decreased in comparison to control (<55%).

Supplementary Table 1: Rh30_A. See Supplementary_Table_1

Supplementary Table 2: Rh30_B. See Supplementary_Table_2

Supplementary Table 3: Rh41_A. See Supplementary_Table_3

Supplementary Table 4: Rh41_B. See Supplementary_Table_4

Supplementary Table 5: Rh4_A. See Supplementary_Table_5

Supplementary Table 6: Rh4_B. See Supplementary_Table_6

Supplementary Table 7: Rh18_A. See Supplementary_Table_7

Supplementary Table 8: Rh18_B. See Supplementary_Table_8

Supplementary Table 9: Proteins regulated by the PAX3/FOXO3 fusion gene. See Supplementary_Table_9
